# Prognostic Factors of Locally Advanced Cutaneous Squamous Cell Carcinoma in Head and Neck Region in Transplanted Patients

**DOI:** 10.3390/diagnostics16030404

**Published:** 2026-01-27

**Authors:** Giulianno Molina de Melo, Murilo Catafesta das Neves, Rafael Dias Romero, Marcello Rosano, Rodrigo Tadashi Martines, Roberto Massao Takimoto, Barbara Greggio, Marcel das Neves Palumbo, Fabio Brodskyn, Arthur Paredes Gatti, Luiz Henrique Guilherme, Fernando Walder, Rodrigo Oliveira Santos, Fabiano Mesquita Callegari, Marcio Abrahao, Onivaldo Cervantes

**Affiliations:** 1 Department of Head and Neck Surgery, Beneficencia Portuguesa of Sao Paulo Hospital, São Paulo 01323-000, SP, Brazil; muriloneves@hotmail.com (M.C.d.N.); rafaeldiasromero@gmail.com (R.D.R.); 2 Department of Otorhinolaryngology and Surgery of Head and Neck, Universidade Federal de São Paulo/Escola Paulista de Medicina (UNIFESP/EPM), São Paulo 04023-062, SP, Brazil; mrosano2@yahoo.com.br (M.R.); tdmartines@yahoo.com (R.T.M.); rmtakimoto@gmail.com (R.M.T.); barbaragreggio@gmail.com (B.G.); marcel.palumbo@unifesp.br (M.d.N.P.); fabio.brodskyn@gmail.com (F.B.); apgatti.ccp@gmail.com (A.P.G.); lhguilherme85@gmail.com (L.H.G.); fewalder@mac.com (F.W.); rodrigoorlccp@uol.com.br (R.O.S.); marcioabrahao@uol.com.br (M.A.); ocervantes@uol.com.br (O.C.); 3 Department of Pathology, Universidade Federal de São Paulo/Escola Paulista de Medicina (UNIFESP/EPM), São Paulo 04023-062, SP, Brazil; fabcallegari@uol.com.br

**Keywords:** prognosis, survival analysis, immunosuppression, cutaneous squamous cell carcinoma, transplant recipient, head and neck neoplasms

## Abstract

**Background:** Cutaneous squamous cell carcinoma (CSCC) is the second most common neoplasm in humans and the most frequent in Brazil (80% in the head and neck region, 20% mortality). Brazil is a world leader in organ transplants (more than 30,000 transplants in 2019). The risk of transplant patients (Tx) developing CSCC is 65–250 times higher, with deeper infiltration, advanced stage, higher local recurrence, occult metastases, and worse survival. **Objective:** To investigate the prognostic factors of locally advanced cutaneous squamous cell carcinoma (LACSCC) of the head and neck region in transplant patients. **Methods:** 16-year retrospective, single-center series of patients with LACSCC in the head and neck region who underwent surgical treatment. Clinical and Tx data, clinical/pathological stage, surgical treatment, parotid/regional and distant metastases, recurrence, and survival were analyzed. **Results:** 156 patients were included: 69.2% women, 65.3 years; mean primary size: 4.24 cm, 66% T3/T4 tumors, 71% grade 2/3 differentiation, 20.5% transplant recipients, follow-up: 33.6 months. The most affected regions were malar/nasal (28.8%) and auricular (19.2%). Surgeries included wide resection with reconstruction (58.9%), exenteration (14.1%), and temporalectomy (11.5%). Univariate analysis: Recurrence: immunosuppressor drugs (*p* = 0.009), transplanted (*p* = 0.006), compromised margin (*p* = 0.049); Mortality: immunosuppression (*p* = 0.028), total resection and reconstruction (*p* = 0.013), stage (8ed) III-IV (*p* < 0.001), compromised margin (*p* < 0.001), neck metastasis with extranodal extension (*p* = 0.018). Multivariate analysis: Recurrence: transplanted HR: 3.69 (*p* < 0.001), neck metastasis extranodal extension HR: 5.41 (*p* < 0.001), evolution to distant metastasis HR: 5.27 (*p* < 0.001); Mortality: neck metastasis extranodal extension HR: 1.94, (*p* = 0.032), compromised margins HR: 1.87 (*p* = 0.001). Main surgical procedures: temporalectomy HR: 2.83 (*p* = 0.007), major rhinectomy HR: 2.47 (*p* = 0.005); Worst overall survival: Tx compared to NonTx (*p* = 0.069); Worst survival with recurrence: Tx compared to NonTx (*p* = 0.005). **Conclusions:** The LACSCC and transplanted (immunosuppressed) group present low survival, worse prognosis; The formulation of specific guidelines to standardize treatment and predict outcomes on this population are strictly necessary.

## 1. Introduction

Brazil is emerging as a leader in organ transplantation worldwide. In the first half of 2019, a total of 13,263 transplants were performed in Brazil (67 pancreas–kidney, 19 pancreas, 203 heart, 2964 kidney, 1069 liver, 49 lung, 1780 bone marrow, and 7112 cornea transplants). Among these transplants, 96% are performed within the public health system, making Brazil the country with the largest global public program for organ, tissue, and cell transplants [1].

Cutaneous squamous cell carcinoma (CSCC) is the second most common neoplasia in humans; according to GLOBOCAN, by 2045, 1,650,214 new cases of nonmelanoma skin cancer are expected to occur in very-high-HDI countries, 273.864 new cases in high-HDI countries, 56.785 new cases in medium-HDI countries, and 37.785 new cases in low-HDI countries (2.41 million from 2022 to 2045) [2], and approximately 1.2 million new cases of head and neck cancer are estimated to occur in 2040, with 680,000 deaths [3]. 

In Brazil, a continental country where miscegenation has been ocurred throughtou history by Anlgo-Saxon (English, Dutch, German) and Latin (Italians, Portuguese and Spanish) colonizers. There were an estimated 173.930 new cases (83.770 men and 93.160 women) of nonmelanoma skin cancer, with approximately 2.000 deaths, in 2020 [4]. Approximately 80% of these CSCCs are in the head and neck region and are responsible for 20% of skin cancer deaths.

The most common neoplasms that affect transplanted patients are nonmelanoma skin cancers, mainly CSCC and basal cell carcinoma (BCC), which originate in the spinous and basal layers of the epidermis, both of which are strongly correlated with excess sun exposure in susceptible patients [5]. The most frequent locations of these skin carcinomas are in the head and neck region, in the danger areas called the “H” or “M-mask” areas. BCC is slow to grow and rarely has metastases, unlike CSCC, which can show rapid growth, extensive infiltration, and lymphatic metastases in approximately 10% of immunocompetent patients.

Compared with the general nontransplant population, the risk of transplant patients developing CSCC is approximately 65–250 times greater, while the risk of developing BCC is only 10–16 times greater [6,7]. A transplant patient is defined as any patient who has undergone the replacement of a diseased organ by a healthy organ, which may be a solid or liquid organ, with variants depending on the donor: autologous, isologous, allogenic, xenogenic, living or deceased.

Several other risk factors have been associated with posttransplant skin cancer, including the intensity and duration of immunosuppressive therapy, the type of organ transplanted, the duration of transplantation, and previous genetic factors, among others [8,9,10]. Ultraviolet radiation, the phenotypic and genotypic characteristics of the patient, and geographic location are known risk factors for the development of skin cancer in the general population and transplant recipients [11].

It is well known that cancer is associated with immunosuppressive drugs, and that changes to the immunosuppression drug scheme could reduce the risk of subsequent skin cancers [11]. Ducrox et al. hypothesized that treatment with T cell-depleting antibodies, used either in induction treatment or to reverse acute rejection, favors the occurrence of aggressive SCC after retransplantation, and that azathioprine should be avoided as much as possible [12]. Patients who were heavily immunosuppressed after the first transplantation seem to be at higher risk of aggressive SCC even after a second transplantation.

According to the previous TNM, a high-risk feature is defined as a CSCC that can progress aggressively in transplant patients and patients with immunosuppression (HIV, leukemias, lymphomas, induced drugs, xeroderma, autoimmune diseases). Other high-risk features include a border greater than 20 mm or an area greater than 10 mm, recurrence, a chronic inflammatory process, a previous radiotherapy site, rapid growth, poor differentiation, more aggressive subtypes (adenoid, adenosquamous, desmoplastic, carcinosarcomatous), and perineural, lymphatic, and vascular invasion or depth of invasion greater than 6 mm [13].

Also, high-risk factors for metastasis from CSCC are currently described as follows: size > 2 cm; depth > 2 mm; Clark level ≥ IV; primary site: scalp, auditory pavilion, and lip; recurrence; and histological findings: poor differentiation, perineural invasion, lymphatic invasion, and patient’s immunosuppression [14].

In nontransplanted patients, CSCC in the head and neck region can metastasize to the parotid gland and lymph nodes with a relatively high incidence, occurring in approximately 1% to 5% of all cases of CSCC on the face and neck. Regional metastases can occur up to five years after resection of the primary skin neoplasia [15]. This presentation is better reported in countries with an Anglo-Saxon origin, such as the review by Karia et al., in which the incidence of lymph node metastasis from CSCC was estimated to be 4.2/100,000 in men and 1.5/100,000 in women [16], while there is little data reported from low-income and lower-middle-income countries.

In transplanted patients, the clinical presentation of CSCC is more aggressive, often advanced SCC or locally advanced CSCC (LACSCC), leading to rapid growth, more advanced clinical presentation, and worse prognosis; moreover, immunosuppression, which is most likely caused by drugs after transplantation or other clinical comorbidities and the occurrence of concomitant hematological neoplasms, plays an important role as a risk factor. Overall, LACSCC is a poorly characterized disease with short treatment responses to radiotherapy and systemic therapies [17].

Importantly, with the increased growth of transplants and CSCC around the world, the incidence of CSCC in transplanted patients has increased; however, the incidence of local recurrence has also increased, with compromised margins in up to 40% of patients and hidden regional metastases in 35% and up to 70% of patients who present with lymph node extracapsular extension, indicating poor survival rates (up to 46%) [18,19,20,21,22,23].

Although LACSCC is unfortunately very common in immunosuppressed patients, the literature is lacking a consensus definition, thus leading to mismatched treatment in some cases [24,25,26]. Following a period of confusion in the medical literature, the term immunosuppression was excluded by the AJCC from its most recent TNM 8th edition, despite its worse prognosis [17,24,27].

Such a picture of increased transplants worldwide and LACSCC in immunosuppressed transplant patients justifies clinical research, with better investigation of risk factors for the development of more aggressive disease than in the general nontransplanted population.

For this article, we realized that there is a need for better criteria to define who is a high-risk transplanted patient with LACSCC, and to determine the degree of prognostic factors to influence the outcome, with the goal of obtaining information to plan better treatment.

The objective of this study was to elucidate the prognostic factors of LACSCC of the head and neck region in transplanted patients.

### Objective

The primary objective was to evaluate the prognostic factors, clinical characteristics, overall survival, and disease-free survival (DFS) of patients with LACSCC of the head and neck region who underwent transplantation.

## 2. Materials and Methods

This was a retrospective study with a single-center cohort centered at a teaching hospital; the population definition was all consecutive patients with LACSCC from the head and neck region with or without a history of organ transplantation, from 2009 to 2025, referred to the institution, and submitted to surgical treatment. Approval from the institutional ethical review board was obtained before beginning this project, as relevant data needed to be obtained from pathological specimens and all patients had provided informed consent. The approved ethics committee number was 0905/2015 (CAAE: 48857315.6.0000.5505) in March 2016.

This study was conducted in accordance with the preferred reporting of case series in surgery (PROCESS) criteria following the PROCESS guidelines [28]. The study followed the Guidelines for Cohort Studies in Surgery by Agha et al. [29] and the Standards for Quality Improvement Reporting Excellence Guidelines (SQUIRE 2.0) [30]. The study was in accordance with the Declaration of Helsinki and has been registered under the WHO Universal Trial Number (UTN) number U1111-1249-0028 and the Brazilian Clinical Trials Registry (ReBeC) number RBR-9WT2WJ.

The inclusion factors were all patients with LACSCC of the head and neck region, with or without organ transplantation, who underwent surgical treatment to control and curative disease intent during the mentioned period, and whose anatomopathological reports and clinical follow-up data were obtained from the database of surgeries performed in the head and neck.

The exclusion criteria included patients who were lost to follow-up, those whose medical records were incomplete, those who were not operated on at the institution, those who had no indication for surgery for other reasons, those who had previous irradiation in the head area and neck, those who refused surgery, those who refused to participate in the study, and those who had non-cutaneous SCC.

Patients with LACSCC were defined as those who experienced deep recurrence or multiple local recurrences from CSCC; those who had CSCC invading deep structures on the face and skull (frontal, maxilla, mandible, mastoid, and temporal bone); the eye, orbit and periorbit tissues, the nose and adjacent structures; the paranasal sinuses, ear, and periauricular structures; parotid tissue; the lip and nearby structures; or the scalp, skull, and deep face muscles; those who presented with regional lymphatic chain metastasis but not distant metastasis; those who were potentially curable but required major surgery; and those who were not potentially curable or who were unlikely to be cured with surgery, radiotherapy, or combination treatment (surgery and radiotherapy) but had needed surgery as locoregional disease control as suggested by the current literature [17,27,31,32,33].

The anatomopathological evaluation of tissue samples was conducted according to university standards, where samples were fixed in 10% formaldehyde, embedded in paraffin, microtomed, and stained with hematoxylin-eosin. The review was performed by our Pathology Department, and all the primary skin cancer samples and surgical specimens were reexamined by two experienced pathologists and classified according to the TNM and WHO criteria to complete the final report [34,35,36,37].

After the CSCC cancer diagnosis was confirmed, a description of the patients and tissue samples was recorded, and the variables considered for statistical analysis were sex, age, comorbidities, transplantation type, time of transplants, drugs used for transplant recipients, date of diagnosis, mean time with symptoms, local primary tumor, surgical procedure, surgical complications, parotid status, neck lymph node status, clinical (cTNM) and pathological (pTNM) stages—as defined by the last edition of the American Joint Committee on Cancer [35]—margin status (exiguous, compromised, or free), perineural invasion (PNI), angiolymphatic invasion (ALI), radio and chemotherapy data, recurrence data, distant metastasis data, date of the last consultation, and patient condition at last consultation (survival with disease, death of disease, and survival without disease).

Statistical analyses used the Mann–Whitney nonparametric test for small samples; a two-proportion Z-test to compare the proportion of two variables for large samples with the level of significance; Fisher’s exact test for small samples with the level of significance; the chi-square test with a significance of *p* < 0.05 for comparing percentages (frequencies); logistic regression (Cox regression) to predict the probability of a given variable; univariate and multivariate analyses with a significance of *p* < 0.05; and Kaplan–Meier survival curves.

The variables included overall survival and disease-free survival (DFS), defined as a lack of locoregional recurrence, with a minimum follow-up period of 6 months.

## 3. Results

Between 2009 and 2025, we identified a total of 187 patients with locally advanced nonmelanoma skin carcinoma. Thirty-one patients were lost to follow-up, had incomplete medical records, had other types of tumors, refused surgical treatment, or refused to participate in the study. The final study group consisted of 156 patients, all with LACSCC in the head and neck region.

- *Baseline Characteristics*

Thirty-two patients in the study group underwent transplantation, of whom 90.6% (29/32) underwent kidney transplantation and 9.4% (3/32) underwent liver transplantation.

The total study population was divided into two groups, transplanted (Tx) and nontransplanted (non-Tx), and survival and other variables of interest were compared.

There was an overall prevalence of 69.2% (108/156) of women; the median age was 65.8 years, with an average primary tumor size of 4.24 cm; 103 (66%) had T3/T4 tumors; 70.5% had comorbidities; 32 (20.5%) were transplant recipients, with an average time since transplantation of 11 years. The average follow-up was 33.6 months, and 84.5% of patients underwent wide surgical resection with flap reconstruction, exenteration, and temporalectomy.

Among the patients in the transplanted group, 20 (62.5%) were in the stage III/IV group, whereas 80 (64.5%) were in the nontransplanted group (*p* = 0.519). In the LACSCC scenario, the T1/T2 ratios in the Tx group (13 patients—40.7%) and the T3/T4 ratio (19 patients—59.3%) were not significantly different from those in the non-Tx group (T1/T2 (61–49.2%) and T3/T4 (63–50.8%) (*p* = 0.474).

**Figure 1:**
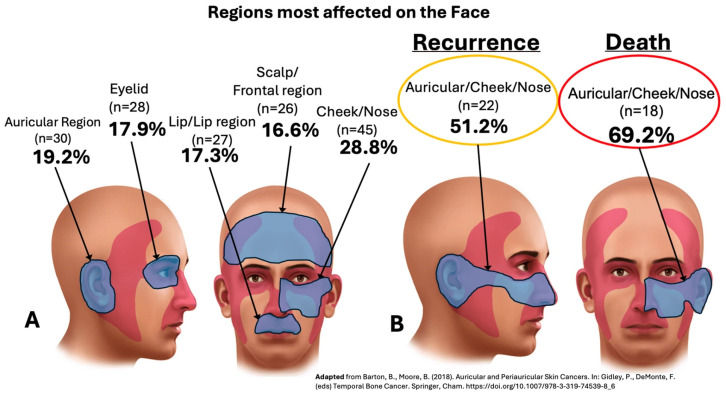
Regions most affected on the face by locally advanced cutaneous SCC (LACSCC), including primary, to recurrence, and to death (**A**,**B**) [38].

Figure 1 shows the regions most affected in the face, leading to either recurrence or death, by the LACSCC in both groups (adapted from Barton et al.) [38].

Table 1 compares the recurrence events according to the quantitative clinical factors.

Table 2 shows the comparison between the endpoint (alive/death) and the quantitative factors of all groups.

**Figure 2:**
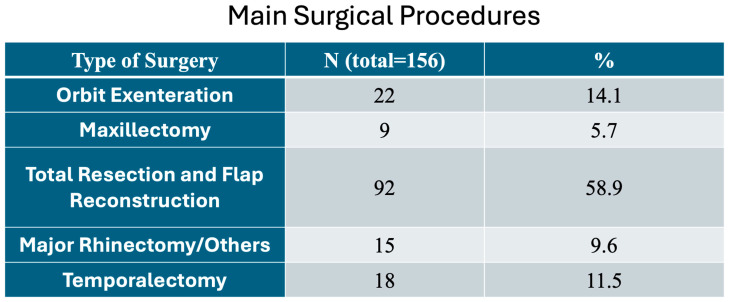
Main surgical procedures performed in the locally advanced cutaneous SCC (LACSCC) patients.

Figure 2 shows the main surgical procedures performed in all patients.

B. *Univariate Analysis*

As shown in Table 3, the univariate analysis revealed that the prognostic factors related to recurrence in all groups were immunosuppressive drugs (*p* = 0.009), transplantation (*p* = 0.006), no facial nerve conservation surgery (*p* = 0.007), compromised margins (*p* = 0.049), distant metastasis (*p* = 0.038), radiotherapy (*p* = 0.027), chemotherapy (*p* < 0.001), and palliative care (*p* < 0.001); the degree of histological differentiation was nearly significant (*p* = 0.051). The main surgical procedures included total primary resection and flap reconstruction (*p* = 0.084), free margins on parotidectomy with neck dissection (*p* = 0.072), and neck metastasis with extranodal extension (*p* = 0.062).

Additionally, in the univariate analysis, the prognostic factors associated with the death endpoint in all groups are shown in Table 4; these factors were significant for mortality in all groups in our cohort: comorbidities (lymphoma) (*p* = 0.028), immunosuppression (*p* = 0.028), primary tumor resection margins (*p* = 0.014), main surgical procedures (total resection and flap reconstruction) (*p* = 0.013), stage (8ed) (IV) (*p* < 0.001), free margins on parotidectomy with neck dissection (*p* = 0.003), compromised margins on definitive surgery (*p* < 0.001), neck metastasis with extranodal extension (*p* = 0.018), distant metastasis evolution (*p* = 0.020), need for radiotherapy (*p* = 0.007), need for chemotherapy (*p* = 0.029), and need for palliative care (*p* < 0.001).

The time interval to regional metastasis was 3 years (6 months to 5 years) since the last recurrence from the primary resection, with a metastatic rate of 100% (84/156 neck dissections with 87 lymph node positive/84 neck dissections, at least 1 lymph node/neck dissection) (Table 3).

We also calculated the lymph node density value as 0.57 (87 lymph nodes positive/152 all lymph nodes), defined as the number of positive lymph nodes divided by the total number of lymph nodes in all groups of neck dissections [39,40,41].

C. *Multivariate Analysis*

Table 5 shows the multivariate analysis results with the hazard ratio for recurrence outcomes in all groups: group (transplanted) HR: 3.69 (*p* < 0.001), evolution to distant metastasis HR: 5.27 (*p* < 0.001), neck metastasis with extranodal extension HR: 5.41 (*p* < 0.001), and nearly no facial nerve conservation HR: 2.95 (*p* = 0.074).

Table 6 shows the multivariate analysis with hazard ratio findings for mortality outcomes in all groups: need for palliative care HR: 0.26 (*p* = 0.001), stage III HR: 0.55 (*p* = 0.003), primary tumor resection margins (compromised) HR: 1.87 (*p* = 0.001), neck metastasis with extranodal extension HR: 1.94 (*p* = 0.032), main surgical procedures (major rhinectomy/others) HR: 2.47 (*p* = 0.005), main surgical procedures (maxillectomy) HR: 3.29 (*p* = 0.041), and main surgical procedures (temporalectomy) HR: 2.83 (*p* = 0.007).

D. *Survival Outcome*

**Figure 3:**
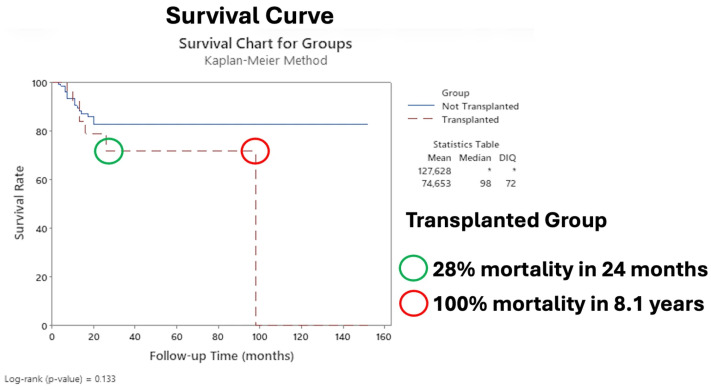
Survival curve comparing transplanted group to nontransplanted group. The asterisk indicates that it was not possible to calculate the statistic.

Figure 3 shows the Kaplan–Meier survival curves comparing the transplanted and nontransplanted groups.

**Figure 4:**
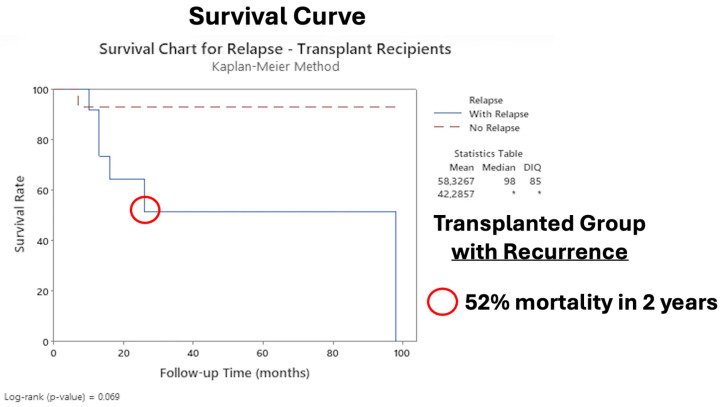
Survival curve of transplanted group comparing recurrence and non-recurrence. The asterisk indicates that it was not possible to calculate the statistic.

Figure 4 shows the Kaplan–Meier survival curves in the transplanted group with recurrence.

## 4. Discussion

CSCC is the second most common neoplasia in humans, with 80% appearing in the head and neck region [3]. The incidence of parotid metastasis ranges from 1% to 5%, and regional metastasis can develop up to 5 years after primary cancer resection in nontransplanted patients [15,16].

In transplanted patients, a population at great risk for the worst progression of CSCC, the risk of developing CSCC is approximately 65–250 times greater, often with more advanced presentation (LACSCC), fast-growing and aggressive behavior, early recurrence, compromised margins (40%), hidden regional metastases (35%), a 70% increase in the number of positive lymph nodes with extracapsular extension, and a decrease in overall survival [6,7,18,19,20,42].

The role of preventive and early intervention measures in most skin cancers is well defined in the literature, but the evidence on cohorts of transplanted patients is still scarce. Preventive measures must always be adopted, like sun protective measures, resections or treatment of all suspected lesions, close follow-up, and prompt aggressive treatment in these patients [13,43]; discussion cases with an experienced team involving the dermatologist, surgeon (ORL-HN), nephrologist, and general physician; adoption of early radiologic exams based on the recurrence suspicion; enhancement of the immunosuppressant drug protocol, with drug changes adapted to each specific case as soon as the index squamous cell carcinoma (SCC) appears; and enhancement of skin monitoring with an expert in the specific ambulatory scenario.

### 4.1. Age, Gender, Follow-Up

In our study, among all LACSCC patients, 69.2% were women, in contrast with the literature [7,44], with a median age of 65.8 years, supported by other studies, with no difference between gender, either in recurrence or mortality (Table 3 and Table 4) [15,19].

Our cohort study revealed 156 patients with LACSCC, all of whom with recurrence from their primary CSCC: 28.8% in the malar/cheek region; 19.2% in the auricular region; 17.9% in the eyelid region; 17.3% in the lip region; and 16.6% in the scalp/frontal region (Figure 1), with a median follow-up of 33.6 months (maximum of 152 months) (Figure 3). Importantly, precisely because we are a referral center, our population is heterogeneous and comes from different states of our country (classified as a developing country). Unfortunately, this could lead to delays in consultations, growth of tumors with aggressive behavior, and further treatment, with the worst outcome observed in the transplanted group (Figure 3). However, as Table 2 showed, a significant *p*-value was found at follow-up, where alive patients lived 33.1 months longer than patients in the death group did (18.2 months) (*p* = 0.03), which can be of important observation since recurrence can influence the outcome.

### 4.2. Stage and Degree of Differentiation

Unfortunately, 66% of our cohort had T3/T4 tumors at the time of the main LACSCC, with an average primary tumor size of 4.24 cm; 70.5% presented with comorbidities, which is a common finding among LACSCC patients and is similar to others studies [11,14,20,42,44]. This particularity could reflect our health system failure, as we have limited health resources and treatment is not as widely available in a continental country as it is in higher income countries, reinforcing the purpose of prognostic factor studies, which will provide important data to create safety pathways for patients and to select adequate treatment [16,45,46,47,48].

Furthermore, our results revealed a 61% prevalence of moderate and undifferentiated histologic differentiation in LACSCC, which was strongly associated with recurrence (*p* = 0.051) (Table 3), a common feature of these advanced neoplasia, and associated with overall poor prognosis, which has been emphasized by other authors [14,24,26,34,45,49].

It is relevant to point out that, similarly to Girardi et al., in comparison to their study of 647 patients in a real-world patient sample, our study found similar results, observing that advanced pT stage was independently associated with poor survival [26]. Another study to identify prognostic factors in CSCC was performed by Zeng et al., who found that poor differentiation, perineural invasion, and a Breslow thickness greater than 2 mm were all associated with an increased risk of metastasis and increased risk of death, demonstrating the consistency of our findings, although we did not use the Breslow classification on our LACSCC cohort, as we used the thickness or depth of invasion definition [13,24,50].

### 4.3. Risk of Metastasis

The reported risk of clinical metastasis from CSCC is 1% to 5%, occurring until five years after primary resection in immunocompetent patients [15,16,49,51]. In contrast, CSCC in transplanted patients is associated with higher rates of local recurrence, with one third presenting hidden regional metastases and 70% presenting lymph node extracapsular extension, with a 46% decrease in survival rates [18,19,21,42,44]. In comparison with other studies, our cohort of patients with LACSCC and neck metastasis showed that the presence of extranodal extension was strongly associated with recurrence (*p* = 0.062) and mortality (*p* = 0.018) according to univariate analysis (Table 3/Table 4); the HR of recurrence was 5.41, and the HR of mortality was 1.94 according to multivariate analysis (Table 5/Table 6). Our findings are similar to those of Caudill et al. and others, who reported that the metastatic rates for these tumors are 25–30% greater than those of nontransplanted patients, which is associated with older age, UV exposure, immune suppression, fair skin, and spread to regional lymph nodes in 80% of cases [42,44,49,52,53].

Our time evolution to metastasis was 3 years (6 months to 5 years), with a metastatic rate of 55.7% (87 positive lymph nodes on neck dissection (ND)/156 total patients in cohort X 100) (Table 3). Interestingly, similar results were reported by McLaughlin et al., who reported a 6-month time frame from resection of the primary lesion to regional lymph node involvement, but in contrast to our data, their metastatic rate was only 5%; this likely reflects the worst clinical status from our transplanted cohort at the time of definitive surgery to LACSCC [44,49,54,55,56].

Our finding of a lymph node density value of 0.57 (87 positive lymph nodes/152 all lymph nodes on ND) (Table 3) proved to be a reliable prognostic factor, as extrapolated data from oral cavity cancer for comparison. In agreement with our results, Liao et al. reported that a lymph node density ≥ 0.05 was associated with poor survival, with an HR of 3.35 (95% CI: 3.05–3.67), and was a significant prognostic indicator of the probability of positive cervical lymph node metastasis [57]. Our findings could help to enhance the risk stratification in prognostic models in LACSCC-transplanted patients; indeed, to date, these findings have not been applied to similar cohorts [39,40,41,57].

### 4.4. Location

In the literature, compared with other body regions, the “H” and “M” areas on the face are at high risk of developing aggressive forms of CSCC for both recurrence and metastasis [5,24], with the lip area being associated with a 5-fold greater risk of nodal metastasis [49,58]. In our LACSCC cases, the most common primary areas were the cheek/nose region, followed by the auricular region and the eyelid and lip region, and those associated with a high risk of recurrence and mortality were the auricular region and cheek/nose region (51.2% and 69.2%, respectively) (Figure 1). Our findings are in line with those in the literature [14,16,18,45,52,59], but unlike some studies, our cohort of patients with primary CSCC face location did not differ in terms of survival according to multivariate analysis; otherwise, we found that the transplanted group variable was the worst predictor of recurrence (*p* < 0.001—HR: 3.69) (Table 5).

### 4.5. Types of Surgery and Outcomes

All surgical plan treatment was based on departmental tumor board discussion, previous patient clinical history, actual primary site, actual TNM and anatomic structures invaded, radiologic exams, associated comorbidities, current literature study, and surgical team experience.

Our main types of surgery were maxillectomy (5.7%), major rhinectomy (9.6%), temporalectomy (11.5%), orbit exenteration (14.1%), and total primary resection with reconstruction (58.9%); 84.5% were submitted to total primary resection and flap reconstruction, orbit exenteration, and temporalectomy (Figure 2).

Among these, the surgeries that could predict mortality outcomes according to multivariate analysis were maxillectomy (HR: 3.29; *p* = 0.041), major rhinectomy (HR: 3.25; *p* = 0.004), and temporalectomy (HR: 3.05; *p* = 0.022) (Table 6). The literature contains abundant data showing that the surgical approach is the most efficient and efficacious course of treatment for patients with CSCC, but few data exist for patients with LACSCC; therefore, our case studies can improve and contribute to the literature in this area [13,60].

In general, the main treatment goal of CSCC is complete removal of the tumor with maintenance of acceptable function and cosmesis [13,24,44,49]. In LACSCC, in addition to surgical technical difficulties, some considerations must be made, such as surgical morbidity, technical feasibility, and the ability to remove tumors with adequate margins. In our LACSCC cohort, all these items were discussed by a multidisciplinary board, also considering the potential for cure/control, patient mental/psychological status, and the ability to heal and tolerate postsurgical alterations, in accordance with some studies and guidelines [13,49,60,61].

Tumor removal by either traditional surgery or Mohs micrographic surgery is the current standard of care for high-risk CSCC [13,24,31,44,52,62], but unfortunately, in our LACSCC cases, this surgery was inadequate, as the majority of cases involved major resection of tissue with skull bone and bones of the face (orbit, maxilla, mandible), cartilage, eyeball, and structures of the nose and ear in a monobloc fashion to achieve the main point of free margins, supported by some studies [31,32]. Indeed, our cohort had an HR of 3.25 (*p* = 0.004) for major rhinectomy surgery, an HR of 3.29 (*p* = 0.041) for maxillectomy, and an HR of 3.05 (*p* = 0.022) for temporalectomy surgery (Table 6). Although the major surgery was intended to be curative, these findings may suggest that performing large surgeries in unfavorable areas of the face in a group of patients with LACSCC who are transplant recipients may not be the best alternative for controlling the disease in this group.

Additionally, our data revealed that immunocompromised patients had the worst prognosis after surgery, with an HR of 3.69 (*p* < 0.001) for recurrence (Table 5), and for immunocompromised patients who experienced recurrence, the probability of mortality was 52% at two years (Figure 4), with 28% mortality at two years compared with that of nontransplanted patients (Figure 3). Our results parallel those of other studies, which reported poorer prognosis in patients with Tx in the immunosuppressed context [7,62,63,64].

### 4.6. Transplanted Group/Immunosuppression Factors

Our cohort included 20.5% of transplanted patients, with a mean age of 59.5 years and average of 11 years of transplantation; most patients had kidney transplants (90.6%) and received mainly tacrolimus and prednisolone drugs (78.1%) (Table 3). Although there was no significant difference between T1/T2 and T3/T4 between nontransplanted and transplanted patients, our findings revealed the worst overall survival in T3/T4, (Figure 3), which is supported by the findings of other authors [5,20,42,62].

As discussed earlier, compared with the general population, organ transplant recipients have a 65 to 250 times greater risk of developing CSCC, and the progression of CSCC is typically more aggressive and less predictable, with greater rates of recurrence and metastasis, leading to more advanced presentation (LACSCC). It presents several poor prognosis factors such as fast growth, early recurrence, hidden regional metastases, and frequent lymph nodes with extracapsular extension, leading to decreased overall survival [6,7,18,19,20,42].

Similarly to our findings, a meta-analysis of five population-based studies, with 31,977 transplant patients, predominantly kidney patients whose follow-up ranged from 6.8 to 8.5 years, demonstrated a 3-fold increased risk of skin cancer in solid organ transplant recipients with aggressive behavior compared with that in the general population [5,65]. Age at the time of transplantation is an important risk factor for the development of posttransplant skin cancers, with a mean of 10–12 years after transplantation, which varies from 6 years (in patients < 50 years of age) to 12 years (>50 years of age), comparable to our cohort data findings of an average age of 59.5 years and a mean of 11 years of transplantation [7,63,66].

Likewise, the duration of immunosuppression and the type of drug used have direct implications for the evolution of LACSCC, as noted in our cohort of mainly tacrolimus and prednisolone users with a long duration of suppression (11 years); these findings are also supported by other studies [12,64,67].

As shown in Table 3, univariate analysis revealed that the prognostic factors related to recurrence in all groups were immunosuppressive drugs (*p* = 0.009) and transplantation (*p* = 0.006), and as shown in Table 4, mortality in all groups was associated with previous comorbidities such as lymphoma (*p* = 0.028) and immunosuppression (*p* = 0.028). Our univariate analysis revealed that immunosuppressed patients are at high risk of either recurrence or mortality due to LACSCC, which is also supported by other studies [5,60,63].

These factors directly affected overall survival in our cohort; we observed 28% and 100% mortality at 2 years and 8.1 years, respectively (Figure 3). Given that there is recurrence in the transplanted patient after the main surgery, a 52% chance of mortality in 2 years occurs compared with that in the nontransplanted group (Figure 4). Our results are worrying, since to date, there is not enough data to guide the treatment of transplanted patients with LACSCC. One possible explanation is that they are excluded from clinical trials (i.e., autoimmune diseases, transplant recipients, patients with immunosuppressive conditions, and those who are being treated with more than 10 mg prednisone) for reasons not adequately described [60,68]. Therefore, our cohort results could help to elucidate and add more data to the literature concerning transplanted patients with LACSCC.

### 4.7. Recurrence and Mortality Factors: Univariate Analysis

The recurrence (primary or regional lymph nodes) of CSCC in the head and neck region is associated with increased risk of lymphatic metastasis, distant metastasis, and mortality [15,27,69,70,71]. Notably, compared with primary CSCC, recurrent CSCC is more aggressive, associated with larger tumor size and deeper infiltration, and has a greater risk of perineural and lymphovascular invasion, as demonstrated in our cases [27,42,60,71,72]. Our cohort was composed of patients with LACSCC, all of whom had recurrence from their primary tumor, with a mean primary tumor size of 4.24 cm and deeper extension to bones (skull and face) and structures (eye, nose, and ear), 55.7% (87 positive lymph nodes/156 patients) of whom had lymph node neck metastasis, and 12.8% (20 positive extranodal extension/156 patients) of whom had extranodal extension (*p* = 0.062) (Table 3), in agreement with the current literature [72].

In the univariate analysis of recurrence (Table 3), our prognostic factors included immunosuppressive drugs (*p* = 0.009), transplantation (*p* = 0.006), no facial nerve conservation surgery (*p* = 0.007), compromised margins (*p* = 0.049), progression to distant metastasis (*p* = 0.038), need for radiotherapy (*p* = 0.027), and chemotherapy (*p* < 0.001). These outcomes were similar to those of some studies that agree that immunosuppression (caused by drugs in patients with Tx), among others, is the worst important prognostic factor in patients with LACSCC [13,24,61,73,74].

When mortality was evaluated by univariate analysis (Table 4), the prognostic factors were previous comorbidities (lymphoma) (*p* = 0.028), immunosuppression (*p* = 0.028), primary tumor resection margins (*p* = 0.014), main surgical procedures (total resection and flap reconstruction) (*p* = 0.013), stage (8ed) IV (*p* < 0.001), free margins on parotidectomy with neck dissection (*p* = 0.003), compromised margins on definitive surgery (*p* < 0.001), neck metastasis with extranodal extension (*p* = 0.018), evolution to distant metastasis (*p* = 0.020), need for radiotherapy (*p* = 0.007), and need for chemotherapy (*p* = 0.029). These outcomes are similar to some reported studies and the study by Clayman et al., who described local recurrence at presentation (*p* = 0.05), invasion beyond subcutaneous tissues (*p* = 0.009), perineural invasion (*p* = 0.002), lesion size (*p* = 0.0003), and depth of invasion (*p* = 0.05) [72].

### 4.8. Multivariate Analysis: Hazard Ratios for Recurrence and Mortality

Our findings of recurrence outcomes in all groups, in multivariate analysis, revealed the following: group (transplanted) HR: 3.69 (*p* < 0.001), evolution to distant metastasis HR: 5.27 (*p* < 0.001), and neck metastasis with extranodal extension HR: 5.41 (*p* < 0.001) (Table 5), which revealed that our cohort of LACSCC patients who underwent transplantation had a high risk of recurrence, lymph node metastasis with extranodal extension, and distant metastasis; some authors have reported similar results, but not in an LACSCC cohort [20,32,42,59,74].

Our important mortality outcomes in all groups were: primary tumor resection margins (compromised) (HR: 1.87; *p* = 0.001), neck metastasis with extranodal extension (HR: 1.94; *p* = 0.032), main surgical procedures (major rhinectomy/others) (HR: 2.47; *p* = 0.005), and main surgical procedures (temporalectomy) (HR: 2.83; *p* = 0.007) (Table 6), which were parallel to the findings of other studies [5,11,24,49,51,52,71]. Recently, a retrospective cohort study by Klein et al. covering 12 international centers, 11,930 patients, and 18,760 tumors (14,766 in immunocompetent patients and 3994 in immunosuppressed patients) has found equivalent results to ours, where immunosuppression was independently associated with local recurrence, distant metastasis, disease-specific death, and major poor outcomes [75].

Interestingly, a systematic review from Zeng et al. with forty-three studies including a total of 21,530 patients and reporting 28,627 cases of CSCC revealed no association between immunosuppression status or location on the cheek and the risk of metastasis or disease-specific death [50].

### 4.9. Kaplan–Meier Survival Curves

Our survival curve revealed 28% mortality at 2 years and 100% mortality at 8.1 years in the transplanted group compared with the nontransplanted group (Figure 3), with a survival of 33.1 months (max 152 months). These results are consistent with the literature, which revealed low survival in the immunosuppressed (Tx) group; thus, our findings reinforce the idea that the group of immunosuppressed individuals—in our study, transplant recipients—has a different biological and humoral response than the nonimmunosuppressed group does, and therefore, more defined and strict criteria should be adopted for the treatment of their primary skin cancers or preventive measures like early immunosuppressive drugs optimization [43,76,77].

Figure 4 shows the survival curve, with 52% mortality at 2 years (8.3 years × 15.6 years) in only the transplanted group: those with recurrence were compared to those without recurrence. These results proved that our selective cohort of LACSCC patients with Tx had a worse prognosis than nontransplanted patients because of several clinical factors discussed above, an expected result since our study data revealed a high risk of recurrence and mortality outcomes in agreement with the literature [24,75,76,77]. Rosenthal et al. studied one thousand and four hundred unique cases of CSCC and reported that immunosuppression was a significant independent predictor of poor outcomes, similar to our findings, but they did not mention any recurrence data [76].

Despite these findings, the NCCN guidelines state that the treatment is primarily based on consensus opinion due limited evidence and that management should prioritize a personalized and multidisciplinary approach over strict adherence to guidelines [43]. We believe that this specific population of LACSCC and immunosuppressed patients deserves further study and individualized treatment, but, if possible, in the form of guidelines, to standardize treatment and predict outcomes, allowing for the allocation of funds and resources to health management entities, one of the contribution of the present article.

We agree with the suggestion that CSCC in immunosuppressed patients, often LACSCC, has a distinct aggressive biology that is not fully understood by traditional staging systems; thus, better criteria are needed to define high-risk LACSCC-transplanted patients and how much prognostic factors influence outcomes in these cohorts. Our work sought to obtain information to determine the best possible treatment for patients with LACSCC, considering all possible biases involved and adding more data to the current literature, with the goal of helping delineate specific guidelines for this population of immunosuppressed patients with LACSCC.

### 4.10. Strengths and Limitations

Our study limitations are those related to retrospective data studies: although the surgical team was the same, selection bias of patients (referred institution) may have occurred; variations in immunosuppressive drug scheme in the transplanted patient group were present; our cohort was heterogenous, with patients coming from different regions of the country, which could have affected the overall result; there was no clinical control group to compare with the surgical patients, as surgery may not be appropriate for all, though this is hard to provide in this very specific cohort; some surgical treatment plans have improved over the years, leading to different results among groups of patients. Our team is highly trained in all these complex procedures; therefore, it may be difficult to reproduce some results in other services.

The strengths of our study were that every patient case was discussed in a multidisciplinary meeting (tumor board) before and after the main surgical plane, diminishing the difference bias between the treatment plan and the surgeon’s preferences, although some differences remain due the variability of LACSCC in these involved populations; this was a centered study, with the same surgical team over the years, highly skilled in these difficult technical procedures.

The complex suite of risk factors for LACSCC in immunosuppressed patients is incompletely understood. However, the current literature supports an etiological role for the intensity and duration of immunosuppression, as well as host factors (age, genetic factors, previous exposures), and our paper played the important role of better characterizing this setting.

## 5. Conclusions

Our results revealed low survival and worse prognosis in the LACSCC and transplanted (immunosuppressed) groups (high risk of recurrence and poor mortality outcomes) because of several clinical factors, reinforcing the idea that immunosuppressed individuals have different biological and humoral responses. The evolution of these patients is not fully understood; therefore, better criteria to define high-risk LACSCC in transplanted patients should be adopted to adequately select the treatment of their skin cancers. Indeed, the formulation of specific guidelines to standardize treatment and predict outcomes in this population is necessary.

## Figures and Tables

**Table 1:** Comparison of recurrence with quantitative factors.

		Mean	Median	Standard Deviation	Coefficient of Variation (CV)	Min	Max	N	IC	*p*-Value
Age	With Recurrence	65.8	65	12.4	19%	34	87	43	3.7	0.952
	Without Recurrence	65.6	67	17.9	27%	23	103	113	3.3	
Primary Cutaneous Tumor Size (cm)	With Recurrence	3.66	3.0	2.17	59%	1.2	10.0	43	0.65	0.897
	Without Recurrence	3.61	3.0	2.13	59%	0.8	10.8	113	0.39	
Neck Dissection	With Recurrence	0.105	0.01	0.192	183%	0.00	0.82	24	0.077	0.490
	Without Recurrence	0.078	0.00	0.145	185%	0.00	0.67	63	0.036	
Years from Transplant	With Recurrence	11.0	11	4.7	43%	3	20	15	2.4	0.267
	Without Recurrence	12.9	11	5.0	38%	3	20	17	2.4	
Follow-up (months)	With Recurrence	33.6	23	34.4	102%	3	151	43	10.3	0.489
	Without Recurrence	29.6	17	30.4	103%	3	152	112	5.6	

**Table 2:** Comparison of endpoint (alive/death) with quantitative factors in the total group.

		Mean	Median	Standard Deviation	Coefficient of Variation (CV)	Min	Max	N	IC	*p*-Value
Age	Death	67.7	70.5	18.8	28%	29	102	26	7.2	0.494
	Alive	65.3	65	16.1	25%	23	103	130	2.8	
Primary Cutaneous Tumor Size (cm)	Death	4.24	4.2	2.10	50%	1.2	10.0	26	0.81	0.103
	Alive	3.50	3.0	2.12	61%	0.8	10.8	130	0.36	
Neck Dissection	Death	0.118	0.00	0.243	206%	0.00	0.82	14	0.127	0.402
	Alive	0.079	0.00	0.138	174%	0.00	0.67	73	0.032	
Years from Transplant	Death	12.0	12	4.6	38%	3	19	8	3.2	0.984
	Alive	12.0	10.5	5.1	42%	3	20	24	2.0	
Follow-up (months)	Death	18.2	11	24.6	135%	3	98	25	9.7	0.030
	Alive	33.1	19.5	32.2	97%	3	152	130	5.5	

**Table 3:** Comparison of recurrence in all groups for distribution of relative frequency of qualitative factors.

		With Recurrence		Without Recurrence		*p*-Value
		N	%	N	%	
Gender	Female	28	65.1%	80	70.8%	0.492
	Male	15	34.9%	33	29.2%	
Comorbidities (Immunosupressor Drugs)	No	17	53.1%	61	78.2%	0.009
	Yes	15	46.9%	17	21.8%	
Group	Not transplanted	28	65.1%	96	85.0%	0.006
	Transplanted	15	34.9%	17	15.0%	
Organ Transplanted	Liver	1	6.7%	2	11.8%	0.411
	Kidney	14	93.3%	15	88.2%	
Transplant Drugs (Tacrolimus)	No	5	33.3%	6	35.3%	0.288
	Yes	10	66.7%	11	64.7%	
Transplant Drugs (Azathioprine)	No	12	80.0%	12	70.6%	0.268
	Yes	3	20.0%	5	29.4%	
Primary Cutaneous SCC Localization	AO	12	27.9%	18	15.9%	0.498
	CF	7	16.3%	19	16.8%	
	LAB	6	14.0%	21	18.6%	
	MMN	10	23.3%	35	31.0%	
	PALP	8	18.6%	20	17.7%	
Degree of Histologic Differentiation	High	8	18.6%	44	38.9%	0.051
	Moderate	24	55.8%	50	44.2%	
	Undifferentiated	11	25.6%	19	16.8%	
Primary Tumor Resection Margins	Compromised	20	46.5%	43	38.1%	0.629
	Exiguous	5	11.6%	15	13.3%	
	Negative	18	41.9%	55	48.7%	
Facial Nerve Conservation	No	3	7.1%	30	27.5%	0.007
	Yes	39	92.9%	79	72.5%	
Main Surgical Procedures	Orbit Exenteration	7	16.3%	15	13.3%	0.084
	Maxillectomy	2	4.7%	7	6.2%	
	Total Resection and Flap Reconstruction	19	44.2%	73	64.6%	
	Major Rhinectomy/Others	6	14.0%	9	8.0%	
	Temporalectomy	9	20.9%	9	8.0%	
Type of Neck Dissection	No	20	46.5%	52	46.0%	0.996
	Radical	10	23.3%	27	23.9%	
	Selective	13	30.2%	34	30.1%	
Lymph Nodes on Neck Dissection	Positive	24	57.1%	63	57.3%	0.988
	Negative	18	42.9%	47	42.7%	
Stage (8ed)	I	4	9.3%	16	14.2%	0.527
	II	7	16.3%	26	23.0%	
	III	17	39.5%	42	37.2%	
	IV	15	34.9%	29	25.7%	
Free Margins on Parotidectomy with Neck Dissection	No	22	51.2%	40	35.4%	0.072
	Yes	21	48.8%	73	64.6%	
Exiguous Margins on Definitive Surgery	No	39	90.7%	100	88.5%	0.693
	Yes	4	9.3%	13	11.5%	
Compromised Margins on Definitive Surgery	No	22	51.2%	77	68.1%	0.049
	Yes	21	48.8%	36	31.9%	
Perineural Invasion	No	29	67.4%	69	61.1%	0.461
	Yes	14	32.6%	44	38.9%	
Angiolymphatic Invasion	No	33	76.7%	92	81.4%	0.513
	Yes	10	23.3%	21	18.6%	
Neck Metastasis with Extranodal Extension	No	34	79.1%	102	90.3%	0.062
	Yes	9	20.9%	11	9.7%	
Evolution to Distant Metastasis	No	35	81.4%	103	92.8%	0.038
	Yes	8	18.6%	8	7.2%	
Need for Radiotherapy	No	9	20.9%	45	39.8%	0.027
	Yes	34	79.1%	68	60.2%	
Need for Chemotherapy	No	26	60.5%	100	88.5%	<0.001
	Yes	17	39.5%	13	11.5%	
Need for Palliative Care	No	26	60.5%	100	88.5%	<0.001
	Yes	17	39.5%	13	11.5%	

Legend: AO: auricular/ear region; CF: scalp/frontal region; MMN: cheek/nose; LAB: lip/lip region; PALP: eyelid/eyelid region.

**Table 4:** Comparison of mortality outcome in all groups to relative frequency distribution of qualitative factors.

		Death		Alive		*p*-Value
		N	%	N	%	
Gender	Female	14	53.8%	94	72.3%	0.063
	Male	12	46.2%	36	27.7%	
Comorbidities (Lymphoma)	No	18	94.7%	91	100%	0.028
	Yes	1	5.3%	0	0.0%	
Group	Not transplanted	18	69.2%	106	81.5%	0.156
	Transplanted	8	30.8%	24	18.5%	
Organ Transplanted	Liver	1	12.5%	2	8.3%	0.445
	Kidney	7	87.5%	22	91.7%	
Transplant Drugs (Tacrolimus)	No	2	25.0%	9	37.5%	0.284
	Yes	6	75.0%	15	62.5%	
Transplant Drugs (Azathioprine)	No	7	87.5%	17	70.8%	0.263
	Yes	1	12.5%	7	29.2%	
Primary Cutaneous SCC Localization	AO	7	26.9%	23	17.7%	0.102
	CF	2	7.7%	24	18.5%	
	LAB	1	3.8%	26	20.0%	
	MMN	11	42.3%	34	26.2%	
	PALP	5	19.2%	23	17.7%	
Degree of Histologic Differentiation	High	5	19.2%	47	36.2%	0.213
	Moderate	14	53.8%	60	46.2%	
	Undifferentiated	7	26.9%	23	17.7%	
Primary Tumor Resection Margins	Compromised	17	65.4%	46	35.4%	0.014
	Exiguous	1	3.8%	19	14.6%	
	Negative	8	30.8%	65	50.0%	
Facial Nerve Conservation	No	4	16.0%	29	23.0%	0.438
	Yes	21	84.0%	97	77.0%	
Main Surgical Procedures	Orbit Exenteration	6	23.1%	16	12.3%	0.013
	Maxillectomy	4	15.4%	5	3.8%	
	Total Resection and Flap Reconstruction	8	30.8%	84	64.6%	
	Major Rhinectomy/Others	3	11.5%	12	9.2%	
	Temporalectomy	5	19.2%	13	10.0%	
Type of Neck Dissection	No	13	50.0%	59	45.4%	0.831
	Radical	5	19.2%	32	24.6%	
	Selective	8	30.8%	39	30.0%	
Stage (8ed)	I	0	0.0%	20	15.4%	<0.001
	II	0	0.0%	33	25.4%	
	III	12	46.2%	47	36.2%	
	IV	14	53.8%	30	23.1%	
Free Margins on Parotidectomy with Neck Dissection	No	17	65.4%	45	34.6%	0.003
	Yes	9	34.6%	85	65.4%	
Compromised Margins on Definitive Surgery	No	8	30.8%	91	70.0%	<0.001
	Yes	18	69.2%	39	30.0%	
Neck Metastasis with Extranodal Extension	No	19	73.1%	117	90.0%	0.018
	Yes	7	26.9%	13	10.0%	
Evolution to Distant Metastasis	No	20	76.9%	118	92.2%	0.020
	Yes	6	23.1%	10	7.8%	
Need for Radiotherapy	No	3	11.5%	51	39.2%	0.007
	Yes	23	88.5%	79	60.8%	
Need for Chemotherapy	No	17	65.4%	109	83.8%	0.029
	Yes	9	34.6%	21	16.2%	
Need for Palliative Care	No	8	30.8%	118	90.8%	<0.001
	Yes	18	69.2%	12	9.2%	

Legend: AO: auricular/ear region; CF: scalp/frontal region; MMN: cheek/nose; LAB: lip/lip region; PALP: eyelid/eyelid region.

**Table 5:** Cox multivariate regression model for predicting recurrence in all groups.

	STEPWISE				
	Coef. (B)	*p*-Value	Hazard Ratio		
			HR	INF	SUP
Group (Transplanted)	1.305	<0.001	3.69	1.84	7.39
Facial Nerve Conservation	1.083	0.074	2.95	0.90	9.67
Neck Metastasis with Extranodal Extension	1.688	<0.001	5.41	2.36	12.40
Evolution to Distant Metastasis	1.662	<0.001	5.27	2.29	12.13

**Table 6:** Cox multivariate regression model for predicting mortality outcome in all groups.

	ENTER					STEPWISE				
	Coef. (B)	*p*-Value	Hazard Ratio			Coef. (B)	*p*-Value	Hazard Ratio		
			HR	INF	SUP			HR	INF	SUP
Neck Metastasis with Extranodal Extension	0.626	0.099	1.87	0.89	3.93	0.664	0.032	1.94	1.06	3.56
Need for Palliative Care	−1.382	0.002	0.25	0.11	0.59	−1.361	0.001	0.26	0.12	0.56
Primary Tumor Resection Margins (Compromised)	0.525	0.024	1.69	1.07	2.67	0.628	0.001	1.87	1.28	2.75
Main Surgical Procedures (Maxillectomy)	1.190	0.041	3.29	1.05	10.31					
Main Surgical Procedures (Temporalectomy)	1.114	0.022	3.05	1.17	7.90	1.041	0.007	2.83	1.33	6.03
Main Surgical Procedures (Major Rhinectomy/Others)	1.180	0.004	3.25	1.46	7.24	0.903	0.005	2.47	1.31	4.66
Stage III	−0.668	0.097	0.51	0.23	1.13	−0.591	0.003	0.55	0.38	0.81

## Data Availability

The data is registered at the WHO Universal Trial Number (UTN) with number U1111-1249-0028.
